# Curcumin suppresses gastric tumor cell growth via ROS-mediated DNA polymerase γ depletion disrupting cellular bioenergetics

**DOI:** 10.1186/s13046-017-0513-5

**Published:** 2017-03-31

**Authors:** Lihua Wang, Xiwen Chen, Zhuanyun Du, Gefei Li, Mayun Chen, Xi Chen, Guang Liang, Tongke Chen

**Affiliations:** 1 Laboratory Animal Centre, Wenzhou Medical University, Wenzhou, Zhejiang China; 2 State Key Laboratory Cultivation Base and Key Laboratory of Vision Science, Ministry of Health of the People’s Republic of China, Zhejiang Provincial Key Laboratory of Ophthalmology and Optometry, Wenzhou, Zhejiang China; 3 School of Ophthalmology and Optometry, Eye Hospital, Wenzhou Medical University, Wenzhou, Zhejiang China; 4 School of Life Sciences, Wenzhou Medical University, Wenzhou, Zhejiang China; 5 Division of Pulmonary Medicine, The First Affiliated Hospital of Wenzhou Medical University, Key Laboratory of Heart and Lung, Wenzhou, China; 6 Chemical Biology Research Center, School of Pharmaceutical Sciences, Wenzhou Medical University, Wenzhou, Zhejiang China; 7 Wenzhou Medical University, University-Town, Wenzhou, Zhejiang 325035 China

**Keywords:** Curcumin, Gastric cancer, Cellular bioenergetics, ROS, POLG

## Abstract

**Background:**

Curcumin, as a pro-apoptotic agent, is extensively studied to inhibit tumor cell growth of various tumor types. Previous work has demonstrated that curcumin inhibits cancer cell growth by targeting multiple signaling transduction and cellular processes. However, the role of curcumin in regulating cellular bioenergetic processes remains largely unknown.

**Methods:**

Western blotting and qRT-PCR were performed to analyze the protein and mRNA level of indicated molecules, respectively. RTCA, CCK-8 assay, nude mice xenograft assay, and in vivo bioluminescence imaging were used to visualize the effects of curcmin on gastric cancer cell growth in vitro and in vivo. Seahorse bioenergetics analyzer was used to investigate the alteration of oxygen consumption and aerobic glycolysis rate.

**Results:**

Curcumin significantly inhibited gastric tumor cell growth, proliferation and colony formation. We further investigated the role of curcumin in regulating cellular redox homeostasis and demonstrated that curcumin initiated severe cellular apoptosis via disrupting mitochondrial homeostasis, thereby enhancing cellular oxidative stress in gastric cancer cells. Furthermore, curcumin dramatically decreased mtDNA content and DNA polymerase γ (POLG) which contributed to reduced mitochondrial oxygen consumption and aerobic glycolysis. We found that curcumin induced POLG depletion via ROS generation, and POLG knockdown also reduced oxidative phosphorylation (OXPHOS) activity and cellular glycolytic rate which was partially rescued by ROS scavenger NAC, indiating POLG plays an important role in the treatment of gastric cancer. Data in the nude mice model verified that curcumin treatment significantly attenuated tumor growth in vivo. Finally, POLG was up-regulated in human gastric cancer tissues and primary gastric cancer cell growth was notably suppressed due to POLG deficiency.

**Conclusions:**

Together, our data suggest a novel mechanism by which curcumin inhibited gastric tumor growth through excessive ROS generation, resulting in depletion of POLG and mtDNA, and the subsequent disruption of cellular bioenergetics.

## Background

Gastric cancer is the fourth most common cancer and the second most frequent cause of cancer death worldwide [1]. Advances in diagnostic and therapeutic approaches have led to excellent expectations for long-term survival for early gastric cancer, whereas the outlook for individuals with advanced gastric cancer is still disappointing [2]. The poor prognosis is frequently explained by lack of early diagnostic biomarkers and effective therapeutic treatments [3, 4]. Thus, there is a high degree of urgency to identify novel more effective therapeutic medicine to overcome this challenge.

Curcumin, a diketone compound isolated from the rhizomes of the plant *Curcuma longa* commonly known as “Haldi” in the Indian subcontinent, is one such agent currently under clinical investigation [5, 6]. The anti-cancer potential of curcumin has been established through multiple animal studies. Curcumin is one of the most successful compounds investigatedin recent years, and is currently being assessed in human both for prevention and treatment of cancer [7–13]. Curcumin exhibits promising pharmacological activities and has demonstrated beneficial effects in terms of cancer cell proliferation, growth, survival, apoptosis, migration, invasion, angiogenesis, and metastasis [14–20]. Several reports have demonstrated that curcumin prevents cancer progression through its anti-inflammatory, antioxidant, anti-proliferative, and pro-apoptotic activities. Although the mechanism of action for this dietary agent has yet to be fully understood, it is believed that curcumin directly interacts with several proteins, including inflammatory molecules, cell survival proteins, histone acetyltransferases(HATs), histone deacetylases (HDAC), protein kinases and reductases, glyoxalase I (GLOI), proteasome, sarcoplasmicreticulum Ca^2+^ ATPase (SERCA), human immune deficiency virus type 1 (HIV1) integrase and protease, DNAmethyltransferases 1 (DNMT1), FtsZ protofilaments, carrier proteins, DNA, RNA, and metal ions [21–25]. Curcumin also affects several transcription factors and co-factors, including nuclear factor-kappa-B (NF-κB) [26–29], activator protein 1 (AP-1) [30], β-catentin [31, 32], signal transducer and activator of transcription3 (STAT3) protein [33, 34], and peroxisome proliferator-activated receptor γ (PPARy) [35, 36]. The effects of curcumin are mediated, at least inpart, through intrinsic and extrinsic apoptosis, p53 [37, 38], NF-κB and NF-κB-regulated gene expression of B cell lymphoma 2 (Bcl2) [39–42], cyclin D1 [36], cyclooxygenase-2 (COX-2) [43], matrix metalloproteinase-9 (MMP-9) [44, 45], Akt [46], mitogen activate protein kinase (MAPK) [47, 48], NF-E2-relatedfactor 2 (Nrf2) [49], and cell–cell adhesion.

Mitochondria have a major role in cellular bioenergetics in most eukaryotic cells, being responsible for producing nearly 95% of cellular ATP through mitochondrial oxidative phosphorylation as well as the control of cell death or survival. Mitochondrial-associated apoptosis is one of the crucial mechanisms of intrinsic cell apoptosis, disruption of mitochondrial homeostasis would lead to initiation of this process. Cellular bioenergetics consists of mitochondrial respiration (OXPHOS) and aerobic glycolysis which contribute to cell growth regulation and other cellular functions. Mitochondria have their own genome known as mtDNA which encodes 13 proteins, 2 rRNAs and 22tRNAs [50]. These 13 mitochondrial proteins are the vital subunits of mitochondrial electron transfer chain complexes in the maintenance of OXPHOS homeostasis. In tumor cells, metabolic reprogramming occurs, resulting in the switch from OXPHOS to aerobic glycolysis to meet the higher energy demands to support the rapid and uncontrolled cell growth, a process known as the Warburg effect [51, 52]. Extensive reports support the view that targeting cellular metabolism could be a promising strategy for cancer treatment. For example, 2-DG disrupts cellular glycolysis [53], mitochondrial glutaminase to inhibit oncogenic transformation [54, 55], AMPK/mTOR axis to suppress tumor cell growth, and AICAR to directly activate AMPK to promote cell cycle arrest and cell apoptosis [56, 57]. Thus, the cellular bioenergetic process is of importance in regulation of cancer cell growth and may be a new strategy for cancer treatment.

To date, the potential role of curcumin in regulation of cellular bioenergetics processes is unclear. In this study, we investigated the hypothesis that curcumin’s anti-cancer effect on gastric tumor cell growth is attributed to disruption of the cellular bioenergetics. Our findings indicated that curcumin could dramatically inhibit gastric tumor cell growth and promoted cell apoptosis. Moreover, we observed that curcumin significantly promoted ROS generation and destroyed cellular bioenergetics, resulting in cancer cell growth inhibition. We further found that curcumin disrupted cellular bioenergetics partially due to ROS-mediated POLG depletion, thereby inhibiting mtDNA replication which further decreased the encoded proteins for energy supplement. These finding in the gastric tumor cells were further supported by studies using animal model and human gastric cancer tissues. In conclusion, we suggest a novel mechanism by which curcumin inhibited gastric tumor growth through excessive ROS generation resulting in depletion of POLG and mtDNA, and the subsequent disruption of cellular bioenergetics.

## Methods

### Reagents and antibodies

Curcumin, oligomycin, carbonylcyanide-p-trifluoromethoxyphenylhydrazone (FCCP), antimycin A, rotenone, glucose were purchased from sigma (St. Louis, MO). Giemsa and crystal violet were purchased from Solarbio Bioscience & Technology (Shanghai, China). MTT assay kit, CCK-8, DCFH-DA ROS detection kit, JC-1 mitochondrial membrane potential detection kit, horseradish peroxidase (HRP)-conjugated anti-rabbit, anti-mouse immunoglobulin G were obtained from Beyotime (Haimen, China). BCA Protein Assay Kit and Pierce ECL Western Blotting Substrate were obtained from Thermo Scientific (Waltham, MA). The monoclonal antibody against β-actin ((#244586) was from Abmart (Shanghai, China). POLG (ab128899), COXI (ab14705), COXII (ab110258), COXIV (ab140643), p-p38T180/Y182 (ab38238), p38 (ab170099) were purchased from abcam (HKSP, New Territories, HK). p21 (#10355-1-AP), ND1 (#19703-1-AP), ND2 (#19704-1-AP) and CytB (#55090-1-AP) were purchased from Protech Group (Wuhan, China). p-ERK^Thr202/Tyr204^ (#9101), ERK (#9102), p-JNK^Thr183/Tyr185^ (#4668), JNK (#9252), phosphor-p53 antibody sampler kit (#9919), Bax (#2774), Bcl-2 (#2870), p-Akt^ser473^ (#4060) and Akt (#9272) were obtained from Cell Signaling Technology. Apoptosis detection assay kit was purchased from BD science.

### Cell culture

Human gastric cancer cell lines, SGC-7901 and BGC-823 were purchased from the Cell Bank of Shanghai Institute of Cell Biology (Shanghai, China).and cultured in RPMI 1640 (Life Technologies) supplemented with 10% fetal bovine serum (FBS) (Life Technologies) and antibiotics (100 U/ml penicillin and streptomycin) at 37 °C in a humidified incubator with 5% CO_2_.

### Colony formation assay

Three hundred SGC-7901 and BGC-823 cells/well were seeded into 6-well plate and cultured at 37 °C with 5% CO_2_. Two weeks later, the cells were washed with pre-warmed PBS for 3 times, fixed with methanol for 20 min, and stained with crystal violet for 15 min. The cells were next washed with ddH_2_O to eliminate residual crystal violet, and colony number was calculated by Image J software.

### Cell proliferation and cell viability assay

For cell proliferation assay, 2 × 10^3^ cells/well were seeded into five 96-well plates, treated with 10 μg/ml of curcumin for 0, 24, 48, 72, or 96 h, and the CCK-8 detection kit was used to determine the relative cell number. h. The OD values at 450 nm were read in a plate reader (Thermo Scientific). Cell viability was assessed using the MTT assay. Six × 10^3^ cells were plated into a 96-well plate, treated with curcumin at 0,2.5,5,10,20, or 40 μg/mL) for 24 h, and assay determined using the MTT kit according to the manufacture’s protocol. In brief, MTT reagent (5 μg/mL) was added to the cells and incubated for 4 h, the crystals produced were dissolved with formazan, and OD values read in plate reader (Thermo Scientific).

### Flow cytometry analysis for apoptosis, ROS determination and mitochondrial membrane potential

For apoptosis analysis, curcumin (10 μg/mL) pre-treated SGC-7901 and BGC-823 cells were washed with ice-cold PBS and collected. Add Annexin V-FITC/PI (BD, San Jose, CA) mixture followed by incubated at room temperature for 20 min protected from light. After that, the samples were subjected to BD Accuri^TM^ C6 flow cytometer (BD, Franklin Lakes, NJ). Intracellular ROS levels were measured as described using the fluorescence probe 2′, 7′-dichlorodihydrofluorescein diacetate (DCFH-DA) according to the manufacturer’s protocol (Beyotime, Shanghai, China). Cells were collected and washed with pre-warmed PBS, followed by incubated with DCFH-DA which dissolved in FBS-free 1640 medium at 37 °C for 20 min. The cells were washed with FBS-free 1640 medium for 2 times and analyzed by FACS. Similarly, mitochondrial superoxide production was determined from the curcumin-treated cells by MitoSOX staining dye according to manufacturer’s instructions, and analyzed by fluorescence microscopy and FACS; *n* = 6. For mitochondrial membrane potential analysis, curcumin (10 μg/mL for 4 h) treated SGC and BGC cells were stained with 2.0 μM JC-1 in complete medium, and incubated for 20 min at 37 °C in the dark. Cells were washed with PBS to remove the excess JC-1 dye, and evaluated by fluorescence microscopy.

### RNA extraction, quantitative real-time PCR and mtDNA determination

Total RNA was extracted from SGC-7901 and BGC-823DNA polymerase γ knockdown cells according to the manufacturer’s protocol. cDNA was synthesized using the PrimeScript^TM^ RT reagent Kit with gDNA Eraser (Takara, Dalian, China). Quantitative real-time PCR assays were performed using 2 μl cDNA/20 μl reaction volumes on a CFX connect^TM^ real-time system (Bio-Rad) using SYBR Green (Bio-Rad) according to the manufacturer’s protocol. Primer sequences are provided in the. For cDNA amplification, the thermal cycling was performed using the following parameters: 95 °C for 10 min, 45 cycles of denaturation at 95 °C for 10 sec and extension at 60 °C for 30 sec. To quantify the mtDNA copy number, genomic DNA was isolated and mtDNA was probed with primers inside ND1 and nuclear DNA with primer inside β-actin. The threshold cycle number (CT) was recorded for each reaction. Each sample was analyzed in triplicate and repeated 3 times. All results are expressed as means ± SD.

### Western blot analysis

Western blot analysis was performed as described previously. In brief, equivalent amount of protein extracts from whole-cell or tissues were separated by SDS-PAGE followed by electrophoretic transfer onto nitrocellulose membrane in Tris-glycine buffer. Block the membrane with 3% non-fat milk in a shaker at room temperature for 1.5 h followed by incubated with indicated primary antibodies at 4 °C overnight. After that, recycle the primary antibodies and wash the membrane 3 × 10 min, and incubated with corresponding secondary antibodies at room temperature for 1 h. Finally, membrane were washed with 1 × TBST for 3 × 10 min and reacted with ECL reagent according to the manufacturer’s protocol (Thermo Scientific, Rockford, IL) for 1 min followed by exposure to X-ray films.

### Measurement of oxidative phosphorylation and glycolysis

Real time integrated cellular oxygen consumption rate (OCR) and extracellular acidification rate (ECAR) were measured using the Seahorse XF96 Extracellular Flux Analyser (Seahorse Bioscience, North Billerica, MA, USA) as previous described. In brief, SGC-7901 or BGC-823 cells were treated with 10 μg/mL curcumin for 12 h and 10^3^ cells were plated into the seahorse customized cell plates. After the probes were calibrated, the OCR was detected with sequential injection of the following compounds which regulate mitochondrial respiration: oligomycin (ATP synthase inhibitor; 1 μM), FCCP (uncoupler; 1 μM), rotenone (complex I inhibitor; 1 μM), and antimycin A (complex III inhibitor; 1 μM). Cellular aerobic glycolysis profile was determined by measuring ECAR from SGC-7901 or BGC-823 cells treated with curcumin. Measurements were made after sequential injection of glucose (10 mM), oligomycin (1 μM), or 2-DG (100 mM); *n* = 6.F. Basal cellular glycolytic rate in SGC-7901 and BGC-823 cells treated with or without 10 μg/mL curcumin. G. Cellular spared glycolytic capacity alteration of curcumin treatment in SGC and BGC cells.

### Nude mice xenograft assay

BALB/c-nu/nu mice (6–8 wk old, female, ~20 g bodyweight) were purchased from Wenzhou Medical University laboratory animal center and fed under specific pathogen-free conditions. All experimental protocols and animal care were compiled with the Guide for the Care and Use of Laboratory Animals, Institute of Laboratory Animal Resources, and were approved by the Institutional Animal Care and Use Committee of Wenzhou Medical University. The nude mice were subcutaneously inoculated with 5 × 10^6^ cells of a 0.1 ml BGC cell suspension. And the tumor nodules were allowed to grow to a volume of ~100 mm^3^ before initiating treatment. The mice were randomized into three groups (six mice for each group). The three groups were intraperitoneally injected with normal saline (control), PBS and 25 mg/kg of curcumin water suspension, respectively. The body weight and tumor volume of each mouse were measured every 2 days over a period of 14 day. The tumor volume was calculated using the following equation: tumor volume = length × width × width/2. On day 14, the mice were sacrificed by overdose of sodium pentobarbital, and its tumor tissues were immediately harvested for further analysis.

### Statistical analysis

All the statistical analysis was performed using SPSS 16.0 (SPSS Standard version 16.0, SPSS Inc., Chicago, IL). Student’s *t* test was used to compare data of different groups. The data are presented as the mean ± standard deviations (SD) values obtained from at least three independent experiments. *P*-values <0.05 was considered statistically significant.

## Results

### Curcumin inhibits gastric tumor cell growth and promotes ROS-mediated apoptosis

**Fig. 1:**
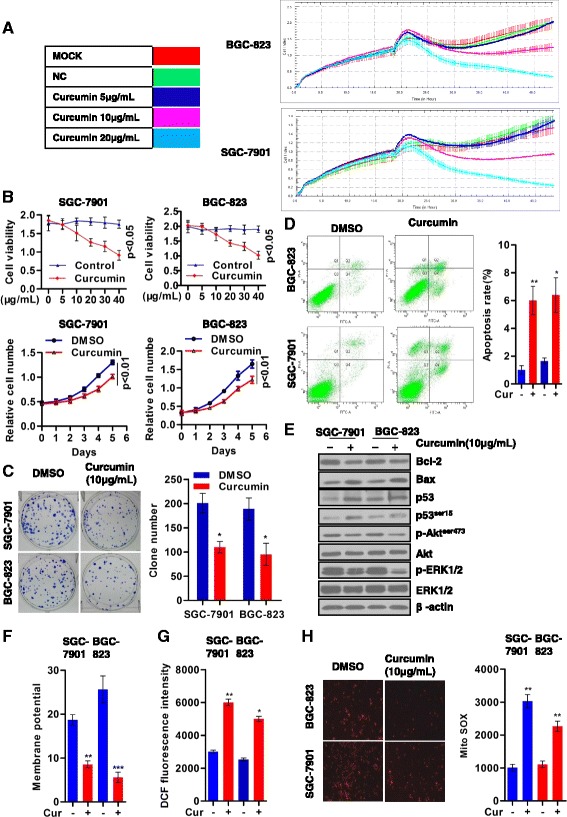
Curcumin inhibits gastric tumor cell growth in vitro and promotes apoptosis. **a**. RTCA was performed to determine the overall cell proliferation curve; *n* = 3. **b**. For cell viability determination, 6 × 10^3^SGC-7901 or BGC-823 cells were seeded into 96-well plates, treated with curcumin at 0,2.5,5,10,20,or 40 μg/mL) for 24 hand assayed based on MTT (Methods) For determination of proliferation, 2 × 10^3^ SGC-7901 or BGC-823 cells were seeded into five 96-well plates, treated with curcumin (10 μg/mL) for 0-5 days and the relative cell number was assayed using the CCK-8 kit (Method).; *n* = 5. **c**. 300 SGC-7901 or BGC-823 cells were cultured in 6-well plates, treated with10 μg/mL curcumin for 5 days, and stained with Giemsa dye for colony formation assessment (Methods); shown are representative dishes of colonies from the treatment groups, and the quantification of the colony number; *n* = 3. **d**. For apoptosis assessment, SGC and BGC cells were treated with 10 μg/mL curcumin for 24 h, prepared using the Annexin V kit (Methods), and analyzed by flow cytometry; shown are representative curves and the quantified data for the experimental groups; *n* = 3. **e**. Representative Western blot analysis of apoptosis-related proteins and signaling molecules of cell lysates from SGC or BGC cells treated with 10 μg/Ml curcumin, β-actin used as loading control; **f**. Mitochondrial membrane potential was determined from SGC or BGC cells treated with10 μg/mL curcumin and the cells prepared for analysis of the fluorescent membrane potential indicator, JC-1 (Methods); *n* = 3. **g**. Intracellular ROS was detected by using the fluorescent probe, DCFH-DA assay kit (Methods). SGC and BGC cells were treated with curcumin (10μg/ml) for 12 h and analyzed by FACs; *n* = 3. **h**. For mitochondrial superoxide production determination, SGC or BGC cells were treated with 10 μg/mL curcumin for 12 h,and were prepared for detection of MitoSOX fluorescence (Methods); *n* = 3. For B-D, F-H, data are presented as mean ± SD; *p < 0.05, **p < 0.01, ***p < 0.001 compared to control or no DMSO group

To investigate the role of curcumin in regulating gastric tumor cell growth, we determined the effects of curcumin concentrations (0-40 μg/ml) on cell viability, and found a dose-dependent reduction of cell viability (Fig. 1a and b). Moreover, curcumin treatment (10 μg/ml) decreased cell proliferation as monitored for up to 5 days (Fig. 1b), significantly suppressed tumor cell colony formation in both SGC-7901 and BGC-823 gastric tumor cells (Fig. 1c). The impact of curcumin in initiating cell apoptosis was evaluated with the PI/FITC dye to identify apoptotic versus non-apoptotic cells by flow cytometry analysis. Results indicated that curcumin treatment (10 μg/ml) of both gastric cell types induced up to 6-fold increase over control of apoptotic cells (Fig. 1d). Current evidence indicated that curcumin likely promoted cell apoptosis via suppressing the Bcl-2 anti-apoptotic pathway as well as inactivating ERK/MAPK signaling. Thus, we asked whether curcumin-mediated gastric tumor cell apoptosis was also dependent on these pathways. Results indicated that curcumin reduced ERK activation and decreased Bcl-2 level which was accompanied with increased p53 serine phosphorylation (Fig. 1e). Figure 1f shows curcumin dramatically disrupted mitochondrial membrane potential, suggesting possible loss of mitochondrial homeostasis (Fig. 1f). Mitochondria contribute to major reactive oxygen species (ROS) generation, which is attributed to the “electron leak” of the electron transport chain (ETC). Although physiological levels of ROS play an important role in normal cell proliferation and cellular signaling, the excessive ROS released from mitochondria can lead to severe oxidative damage and the consequent cell apoptosis. We observed that curcumin treatment of the gastric tumor cells resulted in >2-fold increase in the cellular ROS (Fig. 1g). Significantly, the curcumin-induced increase in ROS content was localized to mitochondria (Fig. 1h). Collectively, our findings indicated that curcumin dramatically promoted cell apoptosis, which was likely through a mechanism of increased mitochondrial oxidative stress.

### Curcumin suppresses mitochondrial respiration and aerobic glycolysis

**Fig. 2:**
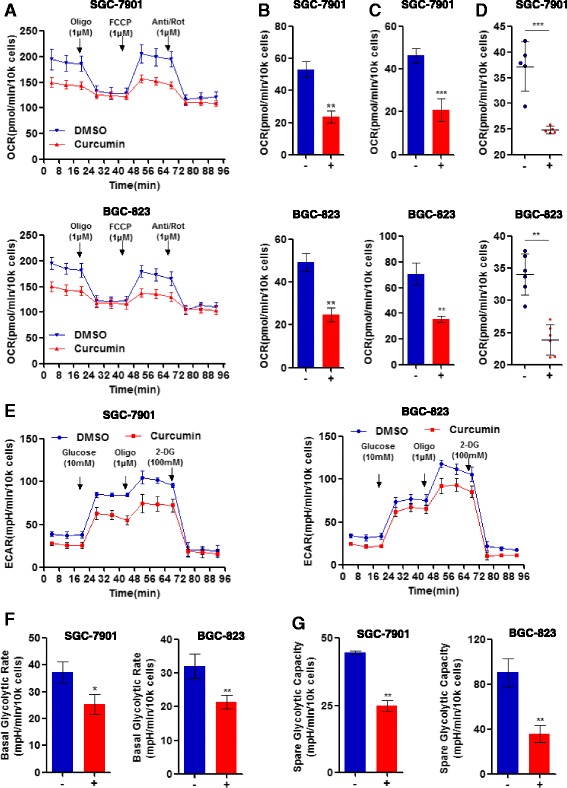
Mitochondrial respiration and aerobic glycolysis are suppressed in response to curcumin. **a**. Effects of curcumin on real-time mitochondrial oxygen consumption rate (OCR). SGC-7901 and BGC-823 cells treated with 10 μg/mL curcumin for 12h were evaluated for mitochondrial oxygen consumption using the Seahorse XF96 analyzer (Methods) after sequential injection (arrows) of the ATP synthase inhibitor oligomycin (1 μM), uncoupler FCCP (1 μM), complex I inhibitor rotenone (1 μM) and complex III inhibitor antimycin A (1 μM); *n* = 6. Effects of curcumin (10 μg/ml for 12 h) on **b**. cellular basal respiration, **c**. cellular maximal respiration rates, and **d**. ATP production of SGC-7901 and BGC-823 cells. **e**. The cellular aerobic glycolysis was evaluated by measures of the extracellular acidification rate (ECAR) from SGC-7901 or BGC-823 cells treated with curcumin (10 μg/ml) following sequential injection (arrow) of glucose (10 mM), oligomycin (1 μM), and 2-DG (100 mM); *n* = 6. The basal (**f**) and spared (**g**) cellular glycolytic rate in SGC-7901 and BGC-823 cells with or without 10 μg/mL curcumin treatment; *n* = 6. Data are presented as mean ± SD; *p < 0.05, **p < 0.01, ***p < 0.001 compared to control or no DMSO group

Mitochondria has a major role in cellular bioenergetics in most eukaryotic cells, being responsible for producing nearly 95% of cellular ATP through mitochondrial oxidative phosphorylation, thereby controlling cell death or survival. We speculated that curcumin would enhance oxidative damage of mitochondrial integrity, which could further limit cellular bioenergetics. To test this hypothesis, we determined the effects of curcumin on mitochondrial respiration (OXPHOS) and aerobic glycolysis using the seahorse 96XF Extracellular Flux Analyser (Methods). As shown in Fig. 2a, curcumin treatment reduced the intact cell oxygen consumption rate (OCR), indicating reduced OXPHOSin SGC-7901 and BGC-823 gastric cancer cells. Furthermore, we assessed specific mitochondrial functions, in particular, basal respiration, maximal respiration and ATP production. As shown in Fig. 2b, curcumin significantly reduced basal respiration which represents the basal mitochondrial OXPHOS activity. Furthermore, in the presence of the uncoupler agent FCCP, cellular oxygen consumption was dramatically increased, while this change was blocked by ETC inhibitors rotenone and antimycin A, inhibiting electron transfer through complex I and complex III. As well, curcumin markedly reduced maximal respiration (Fig. 2c). Integrated mitochondrial electron transfer chain, which drives the H^+^ pump, powers ATP Synthase to catalyze generation of ATP. In SGC-7901 and BGC-823 cells, we found that 10 μg/mL curcumin significantly reduced ATP production (Fig. 2d). These data indicated that curcumin suppressed mitochondrial respiration, resulting in reduced ATP supplement and thereby, restricted cancer cell growth. Otto Warburg had proposed that cancer is the result from the regression of cells to a more primitive metabolism, which is exhibited by proliferating eukaryotic cells, and high aerobic glycolysis may be the most shared metabolic process in cancer cells. We therefore, asked whether curcumin also regulates aerobic glycolysis to limit tumor growth. We determined the effects of curcumin on glycolysis using the seahorse XF96 Extracellular Flux Analyser (Method). Results indicated that curcumin treatment significantly we decreased aerobic glycolysis as measured by extracellular acidification rate (ECAR) in SGC-7901 and BGC-823 cells (Fig. 2e). To further understand the role of curcumin in regulating aerobic glycolysis, we analyzed indices of the basal glycolytic rate and spared glycolytic capacity. As shown in Fig. 2f, curcumin treatment reduced basal glycolytic rate, and together with the observed suppressed mitochondrial respiration, can dramatically inhibit overall tumor cell growth. Moreover, the spared glycolytic capacity was significantly blocked within curcumin (Fig. 2g). Taken together, our findings suggest that curcumin can suppress tumor cell growth likely by limiting cellular bioenergetics.

### Curcumin regulates cellular bioenergetics partially due to POLG depletion and consequent reduced mtDNA content

**Fig. 3:**
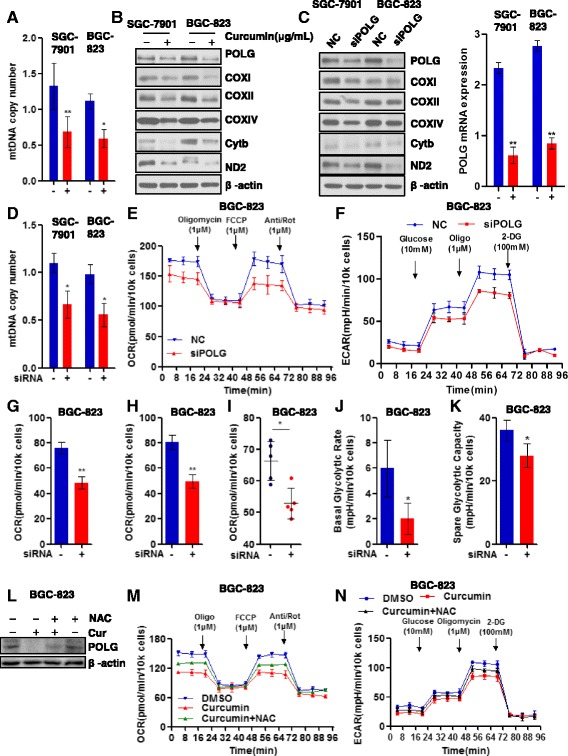
Curcumin down-regulates mtDNA and POLG to inhibit cellular bioenergetics. **a & b** Effects of curcumin (10 μg/ml) on mtDNA copy number and proteins of the respiration complex in SGC-7901 orBGC-823 cells determined by quantitative real-time PCR and Western blot analysis, respectively; shown is representative blot of the respiration complex proteins with β-actin as loading control; curcumin = cur; **c & d** Similar Western blot and Q-PCR analyses were made from SGC-7901 or BGC-823 cells with siRNA-mediated POLG (DNA polymerase γ) knockdown; shown is representative Western blot analysis of respiration proteins; Effects of POLG knockdown in BGC-823 cells on mitochondrial respiration as determined by real-time mitochondrial oxygen consumption rate (OCR), *n* = 6 (**e**) and aerobic glycolysis as determined by extracellular acidification rate (ECAR), *n* = 6 (**f**); arrows indicate sequential injection of respiration complex modifiers as described in Fig. 2. Effects of POLG siRNA-mediated knock-down in BGC-823 cells on **g** basal and **h** maximal respiration as determined by mitochondrial OCR, as well as on **i** ATP production; *n* = 6. Effects of POLG siRNA-mediated knock-down in BGC-823 cells on **j** the basal and **k** spared glycolytic rates, *n* = 6**. l** Shown is representative Western blot analysis of POLG in BGC-823 cells treated N-acetylcysteine (NAC; 10 mM for 4 h) alone or combined with curcumin (cur; 10 μg/ml for 24 min), β-actin as loading control. **m** Effects of NAC (10 mM for 4 h) on the curcumin-induced changes in BGC-823 mitochondrial respiration (OCR), *n* = 6 and **n** aerobic glycolysis (ECAR), *n* = 6; arrows indicate sequential injection of respiration complex modifiers as described Fig. 2. For a, c-k, m-n, data are shown as mean ± SD

Mitochondrial DNA is known to be susceptible to oxidative damage because of a lack of efficient protective mechanisms such as histone protection. We postulated that curcumin-induced elevation of chronic ROS levels could damage mtDNA, leading to disruption of mitochondrial respiration. We investigated this possibility by measuring mtDNA copy number using real-time PCR and found that curcumin treatment resulted in reduced mtDNA level in SGC-7901 and BGC-823 cells (Fig. 3a). Moreover, the curcumin-decreased mtDNA was associated with decreased protein expression of POLG as well as subunits of the respiration complex, such as COXI, COXII, COXIV, CytB (Fig. 3b). POLG is critical for mtDNA replication, transcription and maintenance of mtDNA integrity. To investigate the effects of POLG in modulating mitochondrial respiration in gastric cancer biology, we knockdown POLG using specific target sequences of siRNA targeted which significantly reduced both POLG mRNA and protein levels (Fig. 3c). As expected, POLG knockdown results to reduced expression of subunits of the mitochondrial respiration complex (Fig. 3c). Also, the siRNA-induced knock-down of POLG was associated with reducedmtDNA copy number (Fig. 3d). Based on these findings, we asked whether POLG knockdown also lead to a similar cellular bioenergetics phenotype as that produced by curcumin. Therefore, we determined alterations of OCR (assessment of mitochondrial respiration) and ECAR (assessment of aerobic glycolysis) in gastric cancer cells with siRNA-induced POLG knockdown using the Seahorse 96XF Extracellular Flux Analyser. Results indicated the down-regulated POLG dramatically decrease overall mitochondrial respiration (Fig. 3e) and aerobic glycolysis (Fig. 3f). Additionally, we found that the downregulated POLG was associated with reductions of basal respiration (Fig. 3g), maximal respiration (Fig. 3h), ATP production (Fig. 3i), basal glycolytic rate, (Fig. 3j) and glycolytic capacity (Fig. 3k). We next asked whether excessive ROS generation induced by curcumin was responsible for the POLG depletion. For study, the ROS scavenger, NAC, was used to block curcumin-induced ROS generation in the gastric cancer cells. As predicted, NAC effectively rescued the curcumin-induced POLG depletion as well as the aerobic glycolysis (Fig. 3l-n). Together, our data indicated that curcumin inhibited cancer cell growthvia decreasing POLG, thereby reducing mtDNA content, which further suppressed cellular bioenergetics. These data indicates POLG is significantly involved in the anti-cancer effects of curcumin. Further work is needed to uncover the mechanism by which curcumin downregulates POLG.

### Anti-tumor activity of curcumin in vivo

**Fig. 4:**
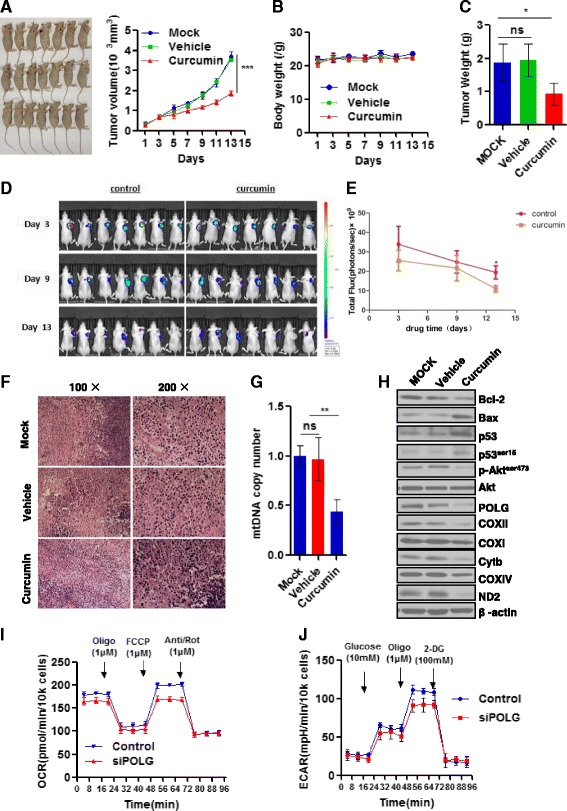
Curcumin suppresses gastric tumor growth in vivo*.* BALB/c-nu/nu were injected subcutaneously with 0.1 ml BGC cell suspension to create a xenograft assay of tumor growth, mock = mock surgery, vehicle = normal saline, curcumin (25 mg/kg), 6 mice/group (Methods); data reported as follows: **a**. Images of nude mice with xenograft showing site of graft. Effects of curcumin on tumor growth is presented as tumor volume changes with time (days). **b**. Mouse body weight. **c**. Tumor weight quantification. **d** & **e**. Live imaging and quantification of gastric tumor cells. **f**. Representative H&E stained histological image of gastric tumor cells. **j**. mtDNA copy number alteration of tumor tissues. **h**.Western blot analysis of apoptosis-related proteins, signaling pathways, and the mitochondrial respiration complex in tumor tissues, β-actin as loading control. The mouse tumors were collected and digested for isolation of primary tumor cells. The POLG was knockdown using siRNA, and cells evaluated for: **i**. mitochondrial respiration as determined by real-time mitochondrial oxygen consumption rate (OCR), *n* = 6 and **j**. aerobic glycolysis as determined by extracellular acidification rate (ECAR), *n* = 6; arrows indicate sequential injection of respiration complex modifiers as described in Fig. 2 (Methods). For A-C, E, G, I-J, data are shown as mean ± SD, *p < 0.05, **p < 0.01 compared to mock or vehicle control

To confirm our data in vivo, we used a xenograft nude mice model in which BGC cells were subcutaneously injected (Methods). Curcumin (25 mg/kg) or normal saline was delivered by IP and tumor size evaluated at 14 days. Results indicated that curcumin caused a progressive decrease in tumor volume as measured for up to 15 days (Fig. 4a). The body weight was not affected by curcumin during this treatment period (Fig. 4b), indicating normal growth condition of the nude mice. As expected, the curcumin-induced decrease in tumor volume was also associated with significant decrease in tumor weight (Fig. 4c). In vivo bioluminescence imaging was used for further confirmation by visualizing and quantification of the effects of curcumin on tumor suppression (Fig. 4d and e). Evaluation of the tumor histology (H&E) indicated that the curcumin-treated group presented with necrosis, resulting in lower cellularity compared to the mock or vehicle controls (Fig. 4f). The mtDNA in tumors from the curcumin-treated group was 50% lower than that of controls (Fig. 4g), which was associated with decreased POLG protein expression as well as the indicated proteins we tested (Fig. 4h). Additionally, the curcumin-treated tumors showed similar changes in expression of apoptosis-related proteins, signaling, and proteins of the respiration complex as those in curcumin-treated gastric cell lines. We next isolated tumor cells from the xenograft nude mice and knocked down POLG with siRNA in these tumor cells. Evaluation of the effects on cellular bioenergetics indicated ECAR and OCR were predictably suppressed (Fig. 4i and j, respectively). Collectively, our in vivo data validate the anti-cancer effects and POLG/cellular bioenergetics-involved mechanism of curcumin.

### Expression of POLG and regulation of cellular bioenergetics in gastric tumor cells from human patients

**Fig. 5:**
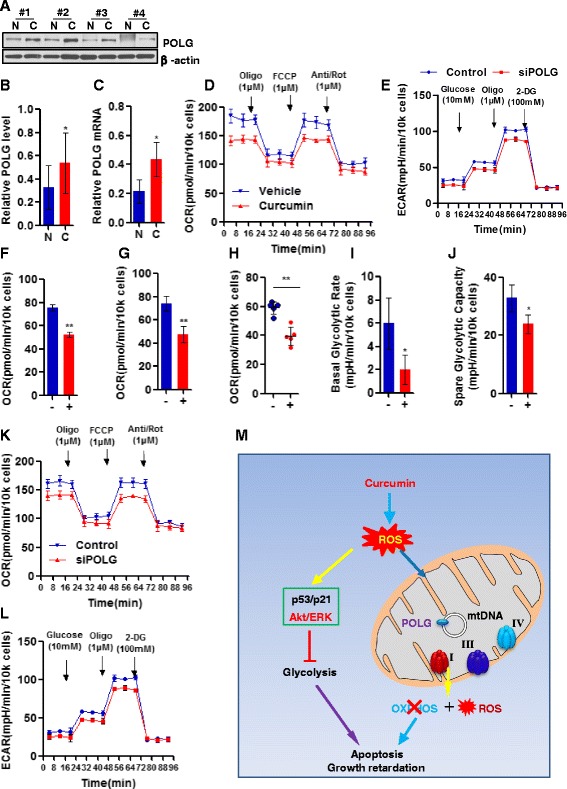
POLG expression in gastric tumors of human subjects and effects of curcumin on the cellular bioenergetics. Gastric cancer (C)and surrounding normal tissues (N) were isolated from human subjects diagnosed with gastric cancer and evaluated for expression of POLG as follows: **a** & **b**. Western blot analysis, β-actin as loading control (*n* = 4)**.** POLG mRNA expression (*n* = 4). The gastric tumor tissues samples were digested for isolation of primary gastric cancer cells for investigation on the effects of curcumin on cellular bioenergetics; **d**. The overall respiration as measured by OCR (mitochondrial oxygen consumption rate), *n* = 6; **e**. Aerobic glycolysis as measured by ECAR (extracellular acidification rate), *n* = 6; **f**. Basal respiration, *n* = 6; **g**. Maximal respiration, *n* = 6; **h**. ATP production; *n* = 6; **i**. Basal glycolytic rate, *n* = 6; and **j**. Spare glycolytic capacity, *n* = 6. The POLG in the human primary gastric cancer cells was knockdown using siRNA, and cells evaluated for: **k**. mitochondrial respiration as determined by real-time mitochondrial oxygen consumption rate (OCR), *n* = 6 and **l**. aerobic glycolysis as determined by extracellular acidification rate (ECAR), *n* = 6; arrows indicate sequential injection of respiration complex modifiers as described in Fig. 2 (Methods) **m**. Schematic working model of curcumin which suppresses gastric tumor cell growth via (i) enhancing excessive ROS generation and p53/p21 activation to initiate apoptosis; and (ii) depleting POLG leading to reduced mtDNA content, impaired OXPHOS, and limited energy supplement. For B-L, data are shown as mean ± SD

We further determined POLG expression in gastric tumors isolated from human subjects diagnosed with gastric cancer, with surrounding normal gastric tissues as comparison. Results indicated that POLG protein and mRNA were dramatically upregulated in tumor tissues compared to normal tissues (Fig. 5a-c). The tumors were digested for isolation of primary tumor cells for investigation of the effects of curcumin on cellular bioenergetics. We observed that curcumin significantly suppressed cellular OXPHOS (Fig. 5d) and glycolysis (Fig. 5e). Furthermore, curcumin decreased the indices of OCR, i.e., the basal respiration, ATP production and maximal respiration (Fig. 5f, g,-h, respectively). And as expected, the parameters of cellular glycolysis, i.e., basal glycolytic rate and spare glycolytic rate were both reduced with curcumin treatment (Fig. 5i and j, respectively). Furthermore, we depleted POLG using siRNA in the primary gastric cancer cells and determined the mitochondrial respiration and aerobic glycolysis. Consistent with data obtained from tumors in the nude mice and the gastric cancer cell lines, POLG depletion resulted in reduced respiration (Fig. 5k) and glycolysis (Fig. 5l). Together, the data obtained from gastric tumors of human subjects further confirmed that curcumin repressed gastric cancer cell bioenergetics.

## Discussion

Curcumin is a promising anti-cancer agent in various types of tumors. Previous evidence indicated that it suppresses and reverts carcinogenesis via multifaceted molecular targets. Several reports have demonstrated that curcumin inhibits animal and human cancers, suggesting that it may serve as a chemo-preventive agent. Numerous in vitro and in vivo experimental models have also revealed that curcumin regulates several molecules in signal transduction pathways including NF-κB, Akt, MAPK, p53, Nrf2, Notch-1, JAK/STAT, β-catenin, and AMPK [26–49]. Modulation of cell signaling pathways through the pleiotropic effects of curcumin likely activate cell death signals and induce apoptosis in cancer cells, thereby inhibiting the progression of disease. However, the role of curcumin in regulating cellular bioenergetics remains unknown. Here, we reported the mechanism by which curcumin regulates mitochondrial respiration and aerobic glycolysis.

Firstly, we found that curcumin treatment led to rapid generation of reactive oxidative species (ROS), enhancing cellular oxidative stress, and thereby leading to cell apoptosis. Faisal Thayyullathil’s work has validated that in addition to caspase 3 activation, curcumin-induced rapid ROS generation leads to AIF release, and the activation of the caspase-independent apoptotic pathway in L929 cells [58, 59]. As reviewed by Paul T, ROS could act like a sword in regulating cancer survival and cell death. Suitable or chronic increased ROS levels can activate signaling pathways in tumor cells, whereas excessive or acute production of ROS may disrupt cell homeostasis via exacerbation of the oxidative damage [60]. These ROS-dependent changes could suppress tumor cell growth or oncogenic transformation.

Mitochondria are the energy factory in the eukaryotic cells producing almost 95% ATP to meet the high energy requirement. In tumor cells, a few reports indicated that mitochondrial respiration remains high in spite of the Warburg effect [51, 52]. We found that curcumin dramatically reduced OCR. Therefore, we asked why curcumin treatment lead to the severe disruption of mitochondrial OXPHOS process. Based on our data, we proposed that the curcumin-induced production of ROS may in turn oxidatively modified enzymes and other proteins of the mitochondrial respiration complex. High glycolytic activity appears to be common in various tumor cells, and targeting this pathway could be a promising way to inhibit cancer cell growth. Our data indicated that curcumin also significantly depressed tumor aerobic glycolysis (Fig. 2). The suppression of glycolysis by curcumin may be related to the acute ROS generation, resulting in phosphorylation of serine 15 of the tumor suppressor protein p53. p53 is reported to play potential roles in modulating cell survival upon DNA damage or hypoxia, as well as functioning as a gene transcription factor [61]. It is also known to be important in several cellular events, such as apoptosis, cell cycle arrest, autophagy, ROS accumulation and metabolism. Here, we found that curcumin activated p53, with potential downstream targets that inhibit tumor growth. Curcumin is reported to increase expression of p53, Bax, MDM2, Bak, PUMA,Noxa, and Bim [62–65], but down-regulate anti-apoptotic factors such asBcl-2, and Bcl-XL [66]. Bensaad and colleagues also reported that p53 may activate TIGAR (an inhibitor of the fructose-2, 6-bisphosphate) to negatively regulate glycolysis [67]. Our results in this study confirmed these previous findings.

Mitochondrial DNA integrity is crucial for mitochondrial homeostasis, and EB-mediated mtDNA depletion lead to severe OXPHOS alteration, resulting in lower cell growth and higher susceptibility to cell stress. We found that curcumin significantly inhibited cellular bioenergetics, which was associated with reduced mtDNA. We suspect that mtDNA is sensitive to oxidative stress, and therefore, the elevated ROS in response to curcumin likely contributed to the reduced mtDNA observed. In addition, our data showed that POLG, a key enzyme participating in mtDNA replication, was dramatically decreased with curcumin treatment. Several reports indicated that POLG plays a critical role in mtDNA replication as well as DNA damage repair. Our finding that curcumin reduced expression of this crucial enzyme suggests that the decreased mtDNA copy number was likely related to the reduced POLG, leading to the subsequent suppression of mitochondrial respiration. Moreover, POLG’s role in tumor cell growth was further supported by the finding that POLG knockdown dramatically delayed cancer cell growth and cellular bioenergetics. As such, the curcumin-induced downregulation of POLG may also inhibited tumor cell growth through in part by decreasing mtDNA and the consequent cellular bioenergetics. Interestingly, POLG was overexpressed in gastric cancer tissues from human subjects, and curcumin inhibited POLG as well as the cellular bioenergetics from the isolated primary tumor cells. However, the mechanism by which curcumin regulates POLG expression level has yet to be defined Data obtained from the tumors in the xenograft nude mouse model provide further confirmation thatcurcumin effectively suppressed gastric tumor cell growth, which was accompanied by reduction of mtDNA and POLG.

## Conclusions

In this study, our findings obtained from gastric cancer cell lines, tumors from xenograft nude mice, and gastric tumors from human subjects, provide strong evidence that curcumin inhibits gastric cancer cell growth via reducing POLG-dependentmitochondrial respiration and cellular aerobic glycolysis. The findings support a novel anti-cancer mechanism of curcumin and provides POLG as a potential target for the treatment of gastric cancer.
